# Gadolinium chloride suppresses acute rejection and induces tolerance following rat liver transplantation by inhibiting Kupffer-cell activation

**DOI:** 10.3892/etm.2014.2015

**Published:** 2014-10-10

**Authors:** YAKUN WU, YUNBING WANG, MIN LI, XIAOLI YANG, JIANPING GONG, WEI ZHANG

**Affiliations:** 1 Department of Hepatobiliary Surgery, Suining Central Hospital, Suining, Sichuan 629000, P.R. China; 2 Department of Hepatobiliary Surgery, The Second Affiliated Hospital, Chongqing Medical University, Chongqing 400010, P.R. China

**Keywords:** gadolinium chloride, Kupffer cells, liver transplantation

## Abstract

The aim of the present study was to investigate the mechanism by which gadolinium chloride (GdCl_3_) inhibits Kupffer cell (KC) activation and its ability to suppress acute rejection and induce tolerance following liver transplantation in rats. Rats were randomly divided into control, liver transplantation with GdCl_3_ pretreatment and liver transplantation with normal saline pretreatment groups. The survival rate, liver function, hepatic pathological histology, cytokine levels in the liver and bile, activity of nuclear factor κ-light-chain-enhancer of activated B cells (NF-κB) in KCs, and expression levels of membranous molecules on the KCs were observed. It was identified that the one-month survival rate in the GdCl_3_ group was significantly higher compared with that in the saline group (P<0.05). The liver function in the GdCl_3_ group gradually recovered following transplantation surgery; however, it progressively deteriorated in the saline group. There were minor changes of hepatic pathological histology in the GdCl_3_ group, whereas changes typical of acute rejection occurred in the saline group. In the GdCl_3_ group, the levels of interferon γ and interleukin (IL)-2 were significantly lower whereas the levels of IL-10 were significantly higher compared with those in the control and saline groups (all P<0.01). The IL-4 levels in the GdCl_3_ and control groups were similar. The activity of NF-κB in the saline group was significantly higher compared with those in the control and GdCl_3_ groups (P<0.01). The expression levels of major histocompatibility complex-II, cluster of differentiation (CD)80 and CD86 on the KC membranes in the GdCl_3_ group was significantly lower compared with those in the control group (P<0.05); however, these membranous proteins were highly expressed in the saline group. These data indicate that GdCl_3_ efficiently inhibits the immunological activity of KCs, suppresses acute rejection and induces tolerance following liver allograft transplantation in rats.

## Introduction

Postoperative acute rejection (AR) in clinical liver transplantation is a major cause of early allograft dysfunction and acute function failure. A number of studies suggest that the inhibitory effect of gadolinium chloride (GdCl_3_) against Kupffer cell (KCs) activation shows potential as a protective intervention in rat models of *in vivo* hepatic reperfusion injury and isolated perfused livers (1–3). It has also been shown that treatment of liver ischemia-reperfusion injury with GdCl_3_ reduces the mortality rate, attenuates neutrophil infiltration and decreases myeloperoxidase activity, improves hepatic function, reduces platelet accumulation in cold perfused livers, and prevents apoptosis of sinusoidal endothelial cells (4). Reduced free radical formation, lipid peroxidation, and parenchymal necrosis following GdCl_3_ administration have also been reported (5). In addition, treatment with GdCl_3_ diminishes the production of reactive oxygen species and the liberation of inflammatory mediators and inhibits the expression of adhesion molecules (6).

Thus, the identification of a treatment that is able to specifically inhibit AR and induce immune tolerance is urgent and essential. The activation of donor KCs is closely correlated with the occurrence of AR with intense phagocytosis, high expression levels of membranous molecules, clearly demonstrated antigen presentation, and the secretion of numerous cytokines that participate in the immune reaction (7–9). Therefore, by blocking the immune activity of KCs, AR may effectively be prevented and inhibited following surgery. In the present study, GdCl_3_ was used to inhibit the immune function of KCs and the depressant function of this treatment on liver transplantation AR was investigated, with the aim of investigating the underlying mechanism and providing experimental evidence for the successful inhibition of postoperative AR in clinical liver transplantation.

## Materials and methods

### Experimental animals and treatment

Male Lewis (LEW) and Brown Norway (BN) rats (210–250 g), used as donors and recipients, respectively, were purchased from the animal research center of Chongqing Medical University (Chongqing, China). All the animals were housed in the animal care facility and received humane care in accordance with the National Institutes of Health guidelines for animal research and the legal requirements in China. The study was approved by the Ethics committee of the Second Affiliated Hospital of Chongqing Medical University (Chongqing, China). All the rats were randomly divided into three groups. The control group comprised BN rats (n=10) that underwent exploratory laparotomy. The GdCl_3_ group (liver transplantation with GdCl_3_ pretreatment group) used LEW (n=15) and BN rats (n=15) and 2 g/l GdCl_3_ solution (7 mg/kg of body weight; Sigma-Aldrich, St. Louis, MO, USA) was injected via the vena caudalis into the donor with two days of continuous administration. On the third day, the donor liver was transplanted to the recipient. The saline group (liver transplantation with normal saline pretreatment group) used LEW (n=15) and BN rats (n=15), and all the operative procedures were just as in the GdCl_3_ group, with the exception of using an identical volume of normal saline instead of GdCl_3_ solution.

### Construction of models of liver transplantation

Orthotopic liver transplantations were performed from the LEW to BN rats using Kamada’s method with a few modifications (10). The infrahepatic vena cava and the portal vein were linked with cuffs. The suprahepatic vena cava was inosculated with suture. A stent tube of the common bile duct was inserted into the common bile duct and the opening of the stent tube was left outside the body and used for collecting bile.

### Analysis of plasma liver function markers and histopathological changes

The recipients were humanely sacrificed for histological inspection seven days post-surgery. The liver tissues were fixed in 100 g/l neutral formalin solution, embedded in paraffin wax and the sections were stained with hematoxylin and eosin to assess the morphological changes. The blood of rats was obtained through the caudal vein. Plasma liver function markers, specifically, serum alanine aminotransferase (ALT), aspartate transaminase (AST) and total bilirubin (TB), were measured with an automatic biochemical meter (Beckman CX7; Beckman Coulter, Brea, CA, USA).

### Reverse transcription polymerase chain reaction (RT-PCR) for cytokine mRNA analysis

RNA was extracted from the liver tissue with a TRIzol reagent kit (Life Technologies, Carlsbad, CA, USA). RT-PCR was performed using an RT-PCR kit (Roche, Los Angeles, CA, USA). The cDNA produced was used for the amplification of interferon (IFN)-γ, IL-2, IL-4, IL-10 and β-actin, respectively (primers were made by the Shanghai Biochemical Products Factory, Shanghai, China). Specific primer sequences of IL-10, IL-2, IL-4, IFN-γ and β-actin were as follows: IL-10, forward: 5′-CCA AGC TTA TCG GAA ATG-3′, and reverse: 5′-CAC TTG TAA ATC TTT CTT CGGG-3′; IL-2, forward: 5′-TAG TGG CTG TCG AGA AGC TGC3′, and reverse: 5′-GGC GTC TTT CAT AGA CAG G-3′; IL-4, forward: 5′-CAT GGT CCG AGA TGT GCA ACT GGC-3′, and reverse: 5′-CGG GCT CAG CAA CTC CAG C-3′; IFN-γ, forward: 5′-CCA CGA GGA ATT CTA CGC CCT GGGC-3′, and reverse: 5′-AAG CTT GGG GAA CAG GTA GG-3′; β-actin, forward: 5′-CAT TGT GAT GGA CTC CGG AG-3′, and reverse: 5′-CTG CCG GTC CAG TAG TATA-3′. The PCR conditions were 30 cycles of denaturation at 94°C for 60 sec, annealing at 58°C for 60 sec and extension at 72°C for 60 sec, and finally an extension at 72°C for 7 min. Agarose gel electrophoresis was used to separate the products of PCR. Ethidium bromide staining, a gelatin imaging system and figure analysis system (GelDoc 2000; Bio-Rad, Hercules, CA, USA) were also used for observing and semiquantitatively counting their relative quantities, expressed as relative optical density (ROD) with normalization to β-actin.

### ELISA for analysis of cytokines in bile

On the seventh day after transplantation, bile was collected in order to measure the expression levels of IFN-γ and IL-4 with an ELISA reagent kit (Beijing Dingguo Changsheng Biotech Co. Ltd., Beijing, China), following the manufacturer’s instructions.

### Isolation of KCs

KCs were isolated using collagenase digestion and differential centrifugation using Percoll. KCs were collected and cultured in plates with RPMI-1640 solution at 37°C in the presence of 5% CO_2_. Non-adherent cells were removed after 6 h by replacing the buffer. The KCs were regulated to a density of 1×10^6^ cells/well. The purity and viability of the cells were >90 and >95%, respectively. Next, the morphological characteristics of the KCs were observed under a phase contrast microscope (BX51; Olympus, Tokyo, Japan).

### Detection of nuclear factor κ-light-chain-enhancer of activated B cells (NF-κB) in KCs

For NF-κB activity analysis, KCs were harvested and lysed at 24 h after liver transplantation, and nuclear proteins were extracted using an Active Motif Nuclear Extract kit (Active Motif, Carlsbad, CA, USA). The relative activity of NF-κB was represented by the optical density value of colorimetric analysis. The DNA-binding activity of NF-κB was determined by ELISA using the TransAM^®^ NF-κB p65 Family kit (Active Motif), according to the manufacturer’s instructions. In brief, nuclear extract was transferred into a 96-well plate coated with NF-κB p65 consensus oligonucleotides. Subsequently, the NF-κB p65 protein bound to the target sequence was detected by primary rabbit anti-rat p65 antibody (Santa Cruz Biotechnology Inc., Santa Cruz, CA, USA) and a goat anti-rabbit horseradish peroxidase-conjugated secondary antibody (Santa Cruz Biotechnology Inc.). Absorbance was quantified by spectrophotometry (DR 5000 spectrophotometer, Hach Company, Indiana, USA) at 595 nm as a relative measure of protein-bound NF-κB p65.

### Membranous molecules on KCs

A monoclonal antibody against major histocompatibility complex (MHC)-II, cluster of differentiation (CD)80 or CD86 (Santa Cruz Biotechnology Inc., Santa Cruz, CA, USA) combined with fluorescein isothiocyanate was added to the liquid suspension of KCs that was acquired previously and the mixture was incubated for 30 min. A flow cytometer (FC500 MPL; Beckman Coulter) was used to assay the positive cells and the mean fluorescence intensity.

### Data analysis

All data are expressed as the mean ± standard deviation. Statistical analyses were performed with SPSS software, version 9.0 (SPSS Inc., Chicago, IL, USA). Analysis of variance with Fisher’s protected least significant difference post hoc analysis and Student’s test was used to identify significant differences between the groups. P<0.05 was considered to indicate a statistically significant difference.

## Results

### Survival condition of postoperative rats

In the initial two days following surgery, all rats in the GdCl_3_ and saline groups survived, and no difference in the quality of life was identified. From the third day following surgery, differences appeared between the groups. Food intake and energy levels gradually recovered to pre-operative levels in the GdCl_3_ group; however, in the saline group, these representations deteriorated. From the seventh day following surgery, the difference became increasingly evident. All the rats in the GdCl_3_ group survived and their general condition recovered to nearly normal levels. However, in the saline group, the rats presented clear occurrence of ascites, jaundice and even mortality. After the first month following surgery, the survival rates in the GdCl_3_ and saline groups were 86 and 47%, respectively, and the difference was statistically significant (P<0.01; Table I).

### Histopathological changes of liver

Under a light microscope, the hepatocytes in the GdCl_3_ group presented moderate vacuolar degeneration and edema and a small number of inflammatory cells were observed to be infiltrating the portal area. However, in the saline group, the hepatocytes presented severe edema and large-area necrosis and large quantities of infiltrating inflammatory cells (not shown).

### Plasma liver function markers

**Figure 1:**
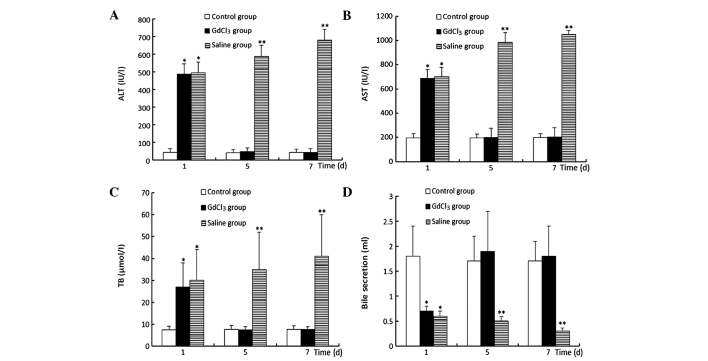
Plasma liver function markers level in the control, GdCl_3_ and saline groups at 1, 5 and 7 days after surgery. (A) ALT, (B) AST and (C) TB levels in the serum. (D) Secretion of bile in the three groups. ^*^P<0.01 vs. control group;^**^P<0.01 vs. control and GdCl_3_ groups. ALT, alanine aminotransferase; AST, aspartate transaminase; TB, total bilirubin.

In the GdCl_3_ group, on the first day following surgery, the ALT, AST and TB levels were clearly elevated and the volume of bile secreted was decreased compared with those in the control group (P<0.05). Notably, from the second day after surgery, the aforementioned markers gradually recovered and by the fifth day after surgery, the markers were restored to normal levels. However, in the saline group, the aforementioned liver function markers were progressively aggravated, increasing to maximum levels on the seventh day following surgery (Fig. 1).

### RT-PCR assay of hepatocellular cytokines

**Figure 2:**
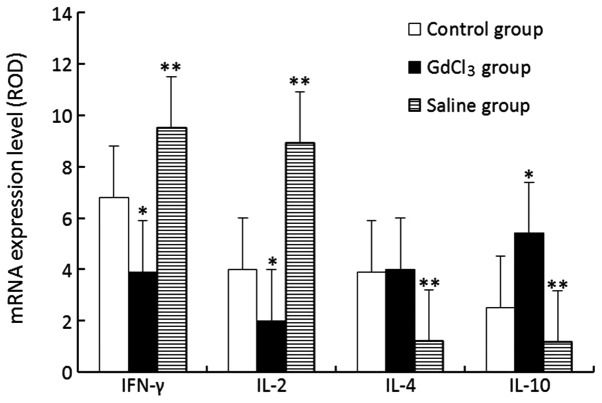
mRNA expression levels of hepatocellular cytokines in the control, GdCl_3_ and saline groups. ^*^P<0.05 vs. control group; ^**^P<0.01 vs. control and GdCl_3_ groups. ROD, relative optical density; IFN, interferon; IL, interleukin.

The expression levels of IFN-γ and IL-2 mRNA in the GdCl_3_ group were evidently lower compared with those in the control group (P<0.05); however, the IL-10 level in the GdCl_3_ group was higher than that in the control group (P<0.05) and the IL-4 levels showed no differences between the GdCl_3_ and control groups. The results for the saline group displayed an opposite trend to those in the GdCl_3_ group (Fig. 2).

### ELISA analytical results of cytokines in bile

**Figure 3:**
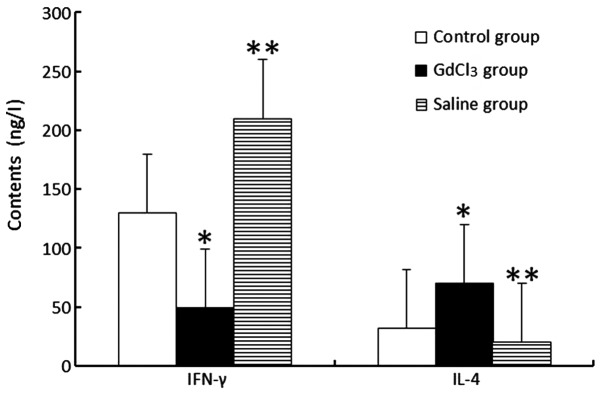
ELISA analytical results of cytokines in the bile. ^*^P<0.05 vs. control group; ^**^P<0.01 vs. the respective control and GdCl_3_ groups. IFN, interferon; IL, interleukin.

In terms of the ELISA analysis of bile, the concentration of IFN-γ in the GdCl_3_ group was markedly lower compared with that in the control group (P<0.05). However, the IL-4 level in the GdCl_3_ group increased compared with that in the control group (P<0.05). The changes observed in the saline groups were completely opposite to those in the GdCl_3_ group (Fig. 3).

### NF-κB activity

**Figure 4:**
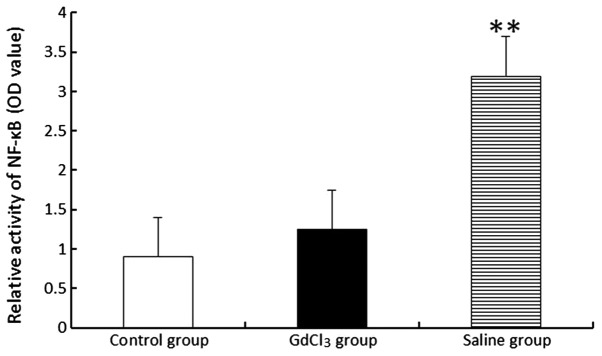
Relative activity of NF-κB represented by the optical density (OD) value in the control, GdCl_3_ and saline groups. ^**^P<0.01 vs. control and GdCl_3_ groups. NF-κB; nuclear factor κ-light-chain-enhancer of activated B cells.

The activity of NF-κB was not revealed to differ between the GdCl_3_ (1.25±0.63) and control (0.91±0.62) groups. However, NF-κB demonstrated an increased activity level in the saline (3.21±0.65) group compared with those in the control and GdCl_3_ groups (P<0.01; Fig. 4).

### Expression levels of membranous molecules on KCs

**Figure 5:**
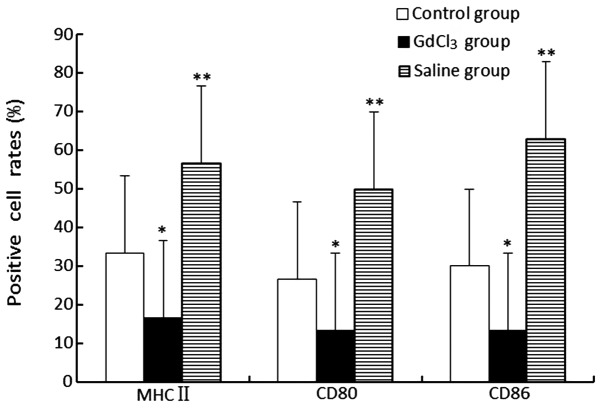
Expression levels of MHC-II, CD80 and CD86 in the control, GdCl_3_ and saline groups. ^*^P<0.05 vs. control group; ^**^P<0.01 vs. respective control and GdCl_3_ groups. MHC-II, major histocompatibility complex class II; CD, cluster of differentiation.

The expression levels of MHC-II, CD80 and CD86 in the GdCl_3_ group were revealed to be lower compared with those in the control and saline groups (P<0.05). Additionally, the saline group highly expressed these three molecules (P<0.01; Fig. 5).

## Discussion

KCs, which are the resident macrophages of the liver, not only exert phagocytosis but also excrete significant amounts of pro-inflammatory and anti-inflammatory cytokines. The understanding of the contribution of KCs to the inflammatory response is likely to help in developing immunological and pharmacological strategies aimed at attenuating the excessive cytokine secretion and decreasing immune damage. Although the role of KCs in directly inducing liver transplantation (LT) tolerance has been reported (7,11), immune tolerance by inhibition of KCs has been highlighted in numerous recent studies (11–13). A number of scholars in China and around the world have demonstrated that GdCl_3_ selectively reduces the number of KCs and their functions in experiments, through an unclear mechanism (3,4,14). Based on this point, if GdCl_3_ was used in a KC-associated study, the results may be beneficial in explaining the mechanism of hepatic postoperative acute rejection. In the present study, the aim was to explore the mechanism of the function of GdCl_3_ with regard to KCs and LT rejection and tolerance, and the following was concluded.

The KCs in the GdCl_3_ group represented a non-activated state with decreased apophysis on the cell surface, lower function of the organelle, and decreased expression of the MHC-II molecule. These observations indicate that GdCl_3_ treatment may inhibit or obstruct phagocytosis, absorption, antigen presentation and the combination capacity of the MHC of KCs with antigens or extraneous materials. In this way, immune rejection may be effectively prevented.

Activated T cells are critical in the immune response, and their activation requires two types of signal stimulus (from the combination of the T-cell receptor and the peptide-MHC and from co-stimulated molecules) (15). As identified in the GdCl_3_ group, the expression levels of co-stimulated molecules (CD80 and CD86) on the KCs decreased. A possible mechanism may be that by downregulating the expression of co-stimulated molecules on KCs, the activation of T lymphocytes at the upper stream was prohibited.

The activation of NF-κB plays a controversial role due to its dual action in the induction of both protective and pro-inflammatory genes. However, it is generally recognized that this dichotomy of NF-κB promoting the hepatic inflammatory response and also protecting against it reflects the expression of NF-κB-dependent genes in different hepatic cell populations (16). In the present study, it was identified that the activation of NF-κB in the group pretreated with GdCl_3_ was lower compared with that in the saline group. Thus, inhibiting the NF-κB activity in KCs may be considered as a route of inducing immune tolerance.

Th0 is able to differentiate into two subsets of helper T cells (Th1 and Th2), depending on the cytokine stimulation and production. Furthermore, the role of KCs in this process cannot be ignored. It is well known that cytokines produced by these two subsets play pivotal roles in the modulation of the immune response. As reported in numerous studies, the Th1 cytokines, including IFN-γ and IL-2, participate in the promotion of graft rejection (16–18). By contrast, an increased expression of the Th2 cytokines, including IL-10 and IL-4, may play a significant role in inducing an immune tolerance (19–24). This study also demonstrated that the IL-10 and IL-4 levels in the group pretreated with GdCl_3_ were evidently higher compared with the respective levels in the saline group; however, the IFN-γ and IL-2 levels in the GdCl_3_ group were clearly lower compared with those in the saline group. These results may indicate that deviation from Th1 to Th2 may be a mechanism of immune tolerance.

In conclusion, GdCl_3_ efficiently inhibited the immunological activity of KCs and suppressed acute rejection following liver allograft transplantation in rats. In addition, it may be concluded that the immune tolerance induced by the inhibitory effect of GdCl_3_ on KCs was a combined action requiring the participation of numerous mechanisms. In further studies, new cytokines and regulative routes could be identified to explain this effect, and further studies may also be necessary for understanding the exact mechanism.

## Figures and Tables

**Table I:** Survival rates of postoperative rats in the control, GdCl_3_ and saline groups.

	Postoperative survival rates, %						
	
Group	1 day	2 days	3 days	4 days	5 days	7 days	1 month
Control	100	100	100	100	100	100	99
GdCl_3_	100	100	100	100	100	100	86^a^
Saline	100	100	93	87	87	80	47

a P<0.01 vs. saline group.
